# On the Use of Aconite as a Therapeutic Agent

**Published:** 1857-12

**Authors:** Edward B. Stevens

**Affiliations:** Cincinnati


*On the use of Aconite as a Therapeutic Agent.* By Edward B. Stevens, M. D., Cincinnati.

I wish to give the readers of this Journal my experiencewith the use of *aconite* as a remedy; especially as I find, in conversation with my medical friends, that very many of them do not avail themselves of an agent, that, with me, has proved very efficient and satisfactory.

The aconitum napellus (monkshood—wolfsbane) is represented by writers, with great uniformity, to rank among our most active poisons; thus, in the article on aconite, in Wharton & Stille's Medical Jurisprudence: "The leaves and root of the aconitum napellus contain one of the most extraordinary and speedy poisons known; and fatal mistakes are recorded from small portions of the leaves or root having been eaten by mistake. Writers also describe the root, seeds, and leaves of the plant as possessing "a hot, acrid taste, giving rise to a burning sensation in the fauces, numbness and tingling in the limbs, swelling and pain in the abdomen, vomiting and purging, accompanied by giddiness, delirium, dimness of sight, and other symptoms indicative of cerebral affection;" and even when simply applied to the cutaneous surface, it is said to produce at first a feeling of heat, which is followed by prickling or tingling sensations, with dumbness.

Although I have directed the use of aconite with comparative frequency, I have not in any case observed such strongly marked manifestations of its therapeutic effect as those which are thus attributed to it; and I am satisfied that, administered *medicinally*, these are exagerated descriptions of its effects.

Another quality attributed to aconite is, that while consciousness may be in no degree impaired, both general and special sensibility will be greatly diminished. Thus, Pereira relates, that under the influence of a full dose, a dog would wag his tail when noticed by his master, and endeavor to follow him around the room, though quite insensible to pinching, or the pricking of a needle.

With such very positive effects upon the nervous system, we should naturally expect this remedy would find a prominent plaee in the treatment of a large class of neuralgic affections, and diseases of a kindred character. It is, however, somewhat singular, that Baron Storck, who first brought aconite into special notice as a remedy, about one hundred years since, more particularly recommended it in scrofula, dropsy, phthisis, and cancer; and having enjoyed a brief but of course fictitious reputation, for the cure of these formidable diseases, it passed for a time almost entirely out of notice again.

I have found good results from the use of aconite, in almost the entire range of neuralgic affections, and in those obscure complications of rheumatism and neuralgia in which there is freedom from local or constitutional trouble, independent of the nervous derangement; but more particularly in such cases as are usually styled pure neuralgia, I have frequently had results almost as prompt and satisfactory as were recently attributed to valerianate of ammonia.

Something more than a year ago, a lady called upon me for advice, for a severe neuralgia in the face and head. From the history of the case, I supposed it to be a result of previous attacks of miasmatic disease, and accordingly prescribed quinine, which relieved her temporarily, but every few weeks she would have a relapse, and for several days at a time suffer excruciatingly. In addition to the quinine, quite a variety of customary treatment was resorted to, until in November I directed the following prescription; after she had suffered a week, and had tried without avail all the remedies that hitherto had given temporary relief:

℞. Tinct. Aconite *Root*, ℨi.,

Tinct. Cimicifuga, ℥ij.

Sig.—To take a teaspoonful every 4 hours.

My patient took three doses, when she was promptly and entirely relieved; and, what is better, she has scarcely felt a neuralgic twinge since, now about ten months.

In another case, a friend had for a long time suffered a peculiar form of neuralgia, or nuuralgic rheumatism in the arm, which seemed to yield to no remedy, even temporarily. I suggested the aconite. The result was equally prompt with that of the case I have just given. I might multiply these satisfactory examples to considerable extent.

The formula given above is the one I most frequently use in administering the aconite to adults. It will be observed that, given in that way, each dose would be equivalent to about 4 drops of the tincture (except that tinct. aconite gives something more than 60 drops to the drachm;) and in that dose I have never seen any effects sufficiently marked or violent to occasion alarm. It will also be observed, that the tinct. of the *root* is directed. The U. S. Dispensatory recognizes two officinal tinctures: of the *leaves* and of the *root.* I prefer the tinct. of the root, as of greater efficiency, and more reliable as to uniformity of effect. The tinct. cimicifuga is intended chiefly as a vehicle, but selected with the view to its contributing to the special effect of the aconite.

I have not tried the aconite in acute rheumatism, but in the chronic rheumatic pains, particularly such as aged people complain of, I have seen very excellent effects. Neither have I used it much locally, though with many, even those who do not use it internally, it is a favorite topical application.

There is a form of neuralgia, associated with uterine derangement, which I have frequently met with, coming up sometimes in connection with the catamenial period, or immediately subsequent to it, in which there is pain through the hips, sacrum, and uterine region. Sometimes I have seen this group of symptoms succeed abortion. I remember a case of this kind, where the local distress I have just alluded to remained very troublesome for several weeks, while much of the time there was almost uninterupted sleeplessness, despite the free use of opiates. The tinct. aconite root, given through the afternoon and evening, releaved the neuralgic pain, and secured a sweet and refreshing sleep through the night.

I have no explanation to give of the *manner* in which aconite acts therapeutically. I offer the suggestion, however, that its action is directly upon the nervous tissue itself. If this be correct, we should find in some sort a key to the irregular action of remedies in neuralgic affections, and some explanation of the fact, that a remedy gives prompt relief in one group of neuralgic cases, while it is apparently void of all effect in a group apparently similar.

These suggestions as to the use of aconite are not given as by any means original, but simply as confirmatory of old known, but not *practically* known truths. The query is naturally suggested, are there not many other active agents sleeping on our shelves, which, if called into service, would materially contribute to the comfort of our patients, and to our own self-satisfaction. Will not, for example, some patient investigator demonstrate that the gungha, or cannabis indica, is a valuable agent, administered *medicinally*, for something more admirable than the unearthly intoxication which is its peculiar manifestation.—*Cin. Med. Obsr.*

